# Articaine as an alternative in lidocaine allergy: Case report of a seventy year old male patient

**DOI:** 10.1016/j.ijscr.2020.11.044

**Published:** 2020-11-11

**Authors:** Mansi Dey, Bibhu Prasad Mishra, Deepti Awasthi, Abhijeeta Sahoo

**Affiliations:** a Oral and Maxillofacial Surgery, ITS Centre for Dental Studies and Research, Muradnagar, Ghaziabad, Uttar Pradesh, India; b Oral and Maxillofacial Surgery, ITS Centre for Dental Studies and Research, Greater Noida, Uttar Pradesh, India; c Oral Medicine and Radiology, ITS Centre for Dental Studies and Research, Muradnagar, Ghaziabad, Uttar Pradesh, India; d Hi-Tech Dental College and Hospital, Bhubaneswar, Odisha, India

**Keywords:** Lidocaine, Allergy, Skin Prick Testing (SPT), Articaine

## Abstract

**Introduction:**

Allergy to lidocaine is extremely rare but if it occurs, one should switch to an alternative drug and discontinue the use of lidocaine so that adverse consequences do not occur.

**Presentation of case:**

We present the case of a 70 year old male patient who had come to our department to undergo extraction of his decayed teeth under local anesthesia.

**Discussion:**

He had history of allergy to lidocaine. Type IV hypersensitivity to lidocaine was confirmed by positive skin prick testing to the drug. Skin prick testing was also performed for articaine that was available with us, and the test was found to be negative without any wheal or flare reaction even after 72 h. Thus it was confirmed that he was non-allergic to articaine and successfully underwent exodontia by using the same.

**Conclusion:**

Articaine can be a suitable alternative in patients with true lignocaine allergy and vice-versa. No cross-reactivity has been reported between lidocaine and articaine so far. However, the number of cases reported in the past are limited, hence more cases are required in the future to prove its authenticity.

## Highlights

• Articaine can be a suitable alternative in patients with true lignocaine allergy.
• Apart from our case report, this fact has also been supported by Khalid Al-Dosary who had reported a case of a 12 year old female patient who was allergic to lidocaine but could tolerate articaine without any cross reactivity between the two drugs.
• On the other hand, few case reports have shown that patients who were allergic to articaine were able to tolerate lidocaine.
• No cross-reactivity has been reported between the two drugs.

## 1 Introduction

Local anaesthesia plays a major role in painless extraction of the teeth. Lidocaine, which is the most common local anaesthetic, has been known to cause allergies or other adverse effects [1,2]. The allergic reactions range from mild symptoms, such as urticaria, erythema, and intense itching, to severe reactions in the form of angioedema and/or respiratory distress. Severe life-threatening anaphylactic responses are in the form of apnea, hypotension, and loss of consciousness [2,3]. Amide-type local anaesthetics are more extensively used as compared to ester-type local anaesthetics, because the latter tend to be more allergenic due to p-aminobenzoic acid (PABA) metabolite that is formed during the degradation process [4]. Patients with genetically abnormal pseudocholinesterase are more likely to suffer from adverse effects of ester local anesthetics while the patients with decreased liver function are more likely to suffer from adverse reactions caused by amide local anaesthetics [5]. We report a case of a patient who was allergic to lidocaine but could tolerate articaine (another amide local anesthetic) that served as an alternative.

## 2 Case report

A 70 year old male patient had reported to our department of Oral and Maxillofacial Surgery for the extraction of his decayed teeth. He had developed itching and hives 24 h after the treatment was done under local anesthesia, in the previous clinic where he had gone to seek treatment.

**Fig. 1:**
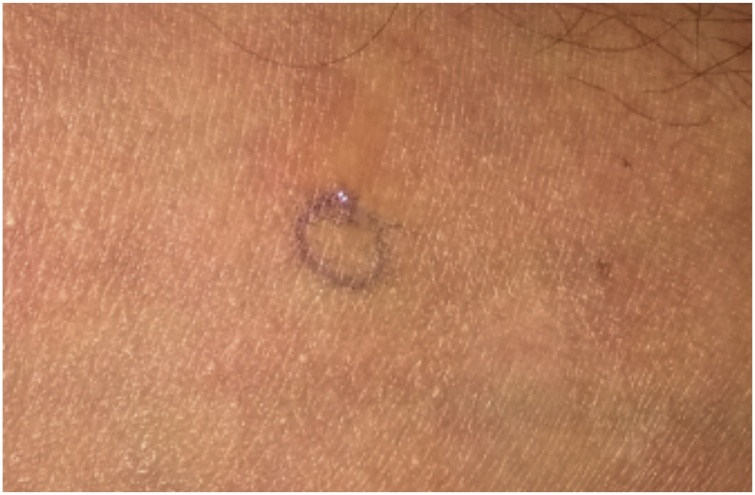
Allergic reaction as a result of type IV hypersensitivity to lidocaine.

Skin prick testing (SPT) was performed with 2% lidocaine. The patient was tested with incremental concentrations of 0.1 mL subcutaneous (SQ) injections. We started with 1:100 dilution, 1:10 dilution, and finally full concentration was injected. A sterile needle was placed through the test solution into the epidermis and gently lifted upward. Each injection was given at an interval of 15 min. The area was evaluated after 10 min for a wheal and flare reaction. The patient did not develop any erythema around the area of injection at that time. He was recalled on the next day and had developed a 6 mm wheal on skin around the site of injection (Fig. 1). Hence the skin test was positive and it was confirmed that the patient had Type IV hypersensitivity to lidocaine.

SPT was then performed for 4% Articaine Hydrochloride. It was performed in the same way as it was performed for lidocaine, and it was found to be negative with 0 mm wheal. The patient was then tested with incremental concentrations of 0.1 mL subcutaneous (SQ) injections. Same as for lidocaine, we started with 1:100 dilution, 1:10 dilution, and finally full concentration was injected. Each injection was given at an interval of 15 min. The patient was recalled for follow-up the next day. There was no reaction to any of the injections after an interval of 24 h. Patient was again recalled on the next two days and reported no allergy. Thus it was confirmed that he was non-allergic to articaine. Hence the patient successfully underwent exondontia using articaine without showing any hypersensitivity reactions.

## 3 Discussion

Local anesthetics are small molecules that induce allergic reactions by acting as haptens, where they bind to an unidentified protein in the serum. Allergic reactions can be of four types based on the immune system's antigen-antibody response [[6], [7], [8], [9]]. Types I, II, and III are immediate-type reactions whereas Type IV is a delayed-type reaction. In Type-I allergic reactions, the first exposure to the sensitizing dose of local anesthetic causes production of immunoglobulin E(IgE) antibody production from B cells without occurrence of any allergic symptom [[7], [8], [9], [10]]. This is followed by binding of the specific IgE antibody to basophils and mast cells. When reexposure to the agent occurs, there is bridging of surface bound antibodies leading to release of inflammatory mediators like histamine from basophils and mast cells [7,9]. Type I reactions manifest as anaphylaxis and can take place immediately, within a few seconds to a few minutes, but the symptoms may take 1–4 h to appear. Symptoms can be limited to the area of skin surrounding the site of administration, in the form of a mild rash, reddening, or urticaria. [7,8,[10], [11], [12]]. Severe generalized reactions may occur involving hypotension, bronchospasm, and cardiac arrest. Type I hypersensitivity reactions can be fatal enough to cause death within minutes of exposure to the offending drug [7,8,12].

In Type II reactions(cytotoxic reactions), IgG and IgM antibodies are primarily involved and are directed against antigens on an individual’s own cells [[7], [8], [9]]. Examples include hemolysis and agranulocytosis. In type III immunologic reactions, antigen-antibody complexes are formed that are not effectively removed by the reticuloendothelial system [[7], [8], [9]], but are deposited in the walls of the blood vessels with subsequent complement fixation causing vascular and connective tissue damage. Type II and Type III reactions have been rarely reported and hence they are not clinically significant with local anesthetics [8]. Type IV reactions are the most prominent with their use [8,9,13]. They involve cellular immunity where T cells are sensitized to the local anesthetic on first exposure, without formation of any antibody. Reexposure to the same local anesthetic causes the memory T cell to release lymphokines that induce inflammatory reactions and activate macrophages to release mediators of inflammatory reactions. Symptoms are similar to Type-1 hypersensitivity reactions but usually take 24–72 h and in some cases just 2 h to manifest.

The reason for increased chances of allergy with the use of ester-type local anesthetics is thought to be their hydrolysis that takes place by cholinesterase, resulting in the release of a metabolite known as para-aminobenzoic acid, which is a known allergen. However, no cases of this phenomenon have been reported by recent studies of ester agents for US Food and Drug Administration approval and marketing claims [[14], [15], [16], [17]]. Allergy caused by lidocaine is rare [1]. It can occur due to the presence of a preservative known as methylparaben, which is a bacteriostatic agent chemically related to para-amino benzoic acid [[18], [19], [20]]. Another similar preservative responsible for allergy by local anesthetics is propylparaben [19,20]. Epinephrine is a vasoconstrictor that is added to the local anesthetics in order to extend their duration of anesthesia. It can cause symptoms like pallor, tachycardia, anxiety, headache, tremor, and hypertension [21]. These symptoms must be distinguished from those caused by lidocaine allergy.

Our reported patient had a classical Type IV hypersensitivity reaction to lidocaine in the form of “anaphylaxis”. Skin prick testing was found to be beneficial in testing the patient for allergy to local anesthetics.

Amide and ester local anesthetics are rarely found to cross react [22]. Amide local anesthetics cross-react with each other occasionally, though less frequently than with esters [23,24]. Similar to our case report, Khalid Al-Dosary had also reported a case of a patient who was allergic to lidocaine but could tolerate articaine without any cross reactivity between the two drugs [25]. On the other hand, few case reports have shown that patients who were allergic to articaine were able to tolerate lidocaine [[26], [27], [28]]. Hence we can say that there is no cross-reactivity between both the drugs. It should also be remembered that we should avoid articaine in those patients who are allergic or hypersensitive to sulphite, because of the presence of sodium metabisulphite as the vasoconstrictor’s antioxidant in it [4]. Also, chances of neurotoxicity are more if articaine is used as a block. Hence it is advisable to administer it in the form of local infiltration. Since not many cases have been reported with the use of articaine as an alternative in lidocaine allergy, we cannot assume it as an only alternative in allergy to lidocaine. Had the patient been allergic to articaine as well, the other options we would have opted for were 1% diphenhydramine, which is a safe and inexpensive method in patients who are allergic to local anesthesics. Another option that we would have opted is general anesthesia. However it is an expensive procedure and not all patients are fit enough to undergo surgery under general anesthesia.

This work has been reported in line with the SCARE 2018 criteria [29].

## 4 Conclusion

Though the lignocaine allergy is rare, detailed medical history should be taken from any patient who is going to undergo treatment under local anesthesia. In case where there are chances of IgE mediated reaction to a local anesthetic, the patient should be tested before carrying out any procedure in order to prevent unwanted consequences. Articaine can be a suitable alternative in patients with true lignocaine allergy and vice-versa. However, due to the limited number of cases that have been reported in the literature, more number of cases are required to be reported in future to prove its authenticity.

## Declaration of Competing Interest

The authors report no declarations of interest.

## Funding

No funding source.

## Ethical approval

Ethical approval was given by Institutional Review Board of ITS Centre for Dental Studies and Research, Muradnagar, Ghaziabad, Uttar Pradesh, India.

## Consent

Written informed consent was obtained from the patient for publication of this case report and accompanying images. A copy of the written consent is available for review by the Editor-in-Chief of this journal on request.

## Author contribution

Concept or design: Dr. Mansi Dey.

Data collection: Dr. Bibhu Prasad Mishra, Dr. Deepti Awasthi, Dr. Abhijeeta Sahoo.

Data analysis: Dr. Mansi Dey, Dr. Bibhu Prasad Mishra, Dr. Abhijeeta Sahoo.

Writing the paper: Dr. Mansi Dey, Dr. Deepti Awasthi.

## Registration of research studies

The following statement applies for all listed authors:

There was no research involving human participants.

There was no trials or observational research undertaken.

This is a case report only.

It has not been reported in man for the first time.

## Guarantor

Dr. Mansi Dey.

## Provenance and peer review

Not commissioned, externally peer-reviewed.
